# Early- and late-life brain reserve proxies and their interactions with education on cognitive outcomes in older adults: A population-based MRI study

**DOI:** 10.1177/13872877261469556

**Published:** 2026-07-20

**Authors:** Xiaojuan Han, Yuanjing Li, Jiafeng Wang, Xinyu Liu, Enming Cao, Yiming Song, Lin Song, Lin Cong, Qinghua Zhang, Shi Tang, Yongxiang Wang, Yifeng Du, Chengxuan Qiu

**Affiliations:** 1 Department of Neurology, 34708 Shandong Provincial Hospital Affiliated to Shandong First Medical University, Shandong University, Jinan, Shandong, P.R. China; 2 Department of Neurology, 518873 Shandong Provincial Hospital, 12589 Shandong University, Jinan, Shandong, P.R. China; 3 Aging Research Center, Department of Neurobiology, Care Sciences and Society, 27106 Karolinska Institute-Stockholm University, Stockholm, Sweden; 4 42750 The Swedish School of Sport and Health Science, GIH, Stockholm, Sweden; 5 Institute of Brain Science and Brain-Inspired Research, Shandong First Medical University & Shandong Academy of Medical Sciences, Jinan, Shandong, China

**Keywords:** Alzheimer's disease, brain reserve, cognitive function, education, population-based study

## Abstract

**Background:**

The interplay between early- and late-life brain reserve (BR) and early-life education in shaping late-life cognitive function requires further elucidation.

**Objective:**

We sought to examine the associations of early- and late-life BR and cognitive function among rural older adults in China, and to assess whether education influenced their relationships.

**Methods:**

In this cross-sectional study (n = 1304), early-life and late-life BR were proxied by total intracranial volume (TIV) and the ratio of total normal brain tissue volume-to-TIV, respectively. Both measures were dichotomized into high and low BR according to their median. Education was classified as high (any formal schooling) and low (no formal schooling). We administered a neuropsychological test battery to assess memory, verbal fluency, executive function, and attention. Mild cognitive impairment (MCI) was diagnosed following Petersen's criteria. Regression analyses were performed.

**Results:**

Higher late-life BR was associated with better performance across all cognitive domains (p < 0.01) and reduced likelihoods of MCI and amnestic MCI (p < 0.001). Joint-exposure analyses suggested that compared to participants with low BR and low education, individuals with both high late-life BR and high education showed best cognitive performance and lowest likelihoods of MCI, followed by those with low BR and high education. Moreover, higher late-life BR was linked to better executive function, attention, and global cognition only in participants with high education (p for interaction<0.05).

**Conclusions:**

Late-life BR has a stronger association with cognitive health than early-life BR, even among older adults without formal schooling. Furthermore, formal education may enhance the association between late-life BR and cognitive function.

## Introduction

Growing evidence has suggested that risk reduction and prevention of dementia may be feasible through interventions targeting modifiable risk factors,^1–3^ such as promoting active lifestyles and controlling cardiovascular risk factors. Adherences to active lifestyles may enhance reserve capacity, a theory that has gained increasing attention. There are two distinct models of reserve theory: brain reserve (BR) and cognitive reserve (CR). The BR model is a passive model of reserve capacity that is determined primarily by structural brain integrity. Current evidence indicates that the human brain stops growing and reaches its maximum volume by the mid-twenties.^4^ Several studies have used head circumference^5^ or total intracranial volume (TIV)^6^ to reflect the static nature of early-life BR. On the other hand, structural brain integrity varies throughout the lifespan due to aging and the accumulation of brain pathology,^7^ resulting in a dynamic BR in later life. Previous research has shown that a high level of BR can delay cognitive decline in individuals at the preclinical stage of dementia and may even offer protection against Alzheimer's disease (AD) symptoms.^5,8^ However, the possible differential influences of early- and late-life BR on cognitive function remain unclear. By contrast, CR is considered an active form of reserve, reflecting the brain's capacity to optimize cognitive function by efficiently regulating neural networks or recruiting compensatory mechanisms when facing aging or brain pathology.^9,10^ Due to the conceptual nature of CR as a hypothetical construct, direct measurement is currently unavailable, and estimation is thus based on a range of proxy indicators. Educational attainment in early life is the most commonly used proxy for CR, which plays a pivotal role in shaping cognitive phenotypes in later life.^11–13^

Numerous epidemiological studies and meta-analyses have linked higher educational attainment to lower likelihoods of cognitive decline, mild cognitive impairment (MCI), and dementia in later life.^14,15^ Nevertheless, the combined impact of BR and educational attainment on cognitive profile remain underexplored in population-based research. Evidence indicates that lower educational attainment is associated with reduced structural brain integrity, such as lower grey matter (GM) volume^16^ and thinner brain cortex,^17^ which could influence BR capacity in later life. Consequently, exploring the relationships between BR at various stages of life and cognitive performance, especially in those with limited educational attainment, may offer important insights into the mechanisms underlying cognitive aging and inform intervention strategies that aim to promote cognitive health in later life.

In this population-based cross-sectional study, we sought to examine the associations of early- and late-life BR with cognitive profile among dementia-free older adults who were living in rural China, and further, to assess the impact of educational attainment on their relationships. We hypothesized that greater late-life BR would be associated with better cognitive performance in older age, and that this association would be moderated by early-life educational attainment such that individuals with both high BR and high education would exhibit the highest cognitive performance.

## Methods

### Study design and participants

**Figure 1.**
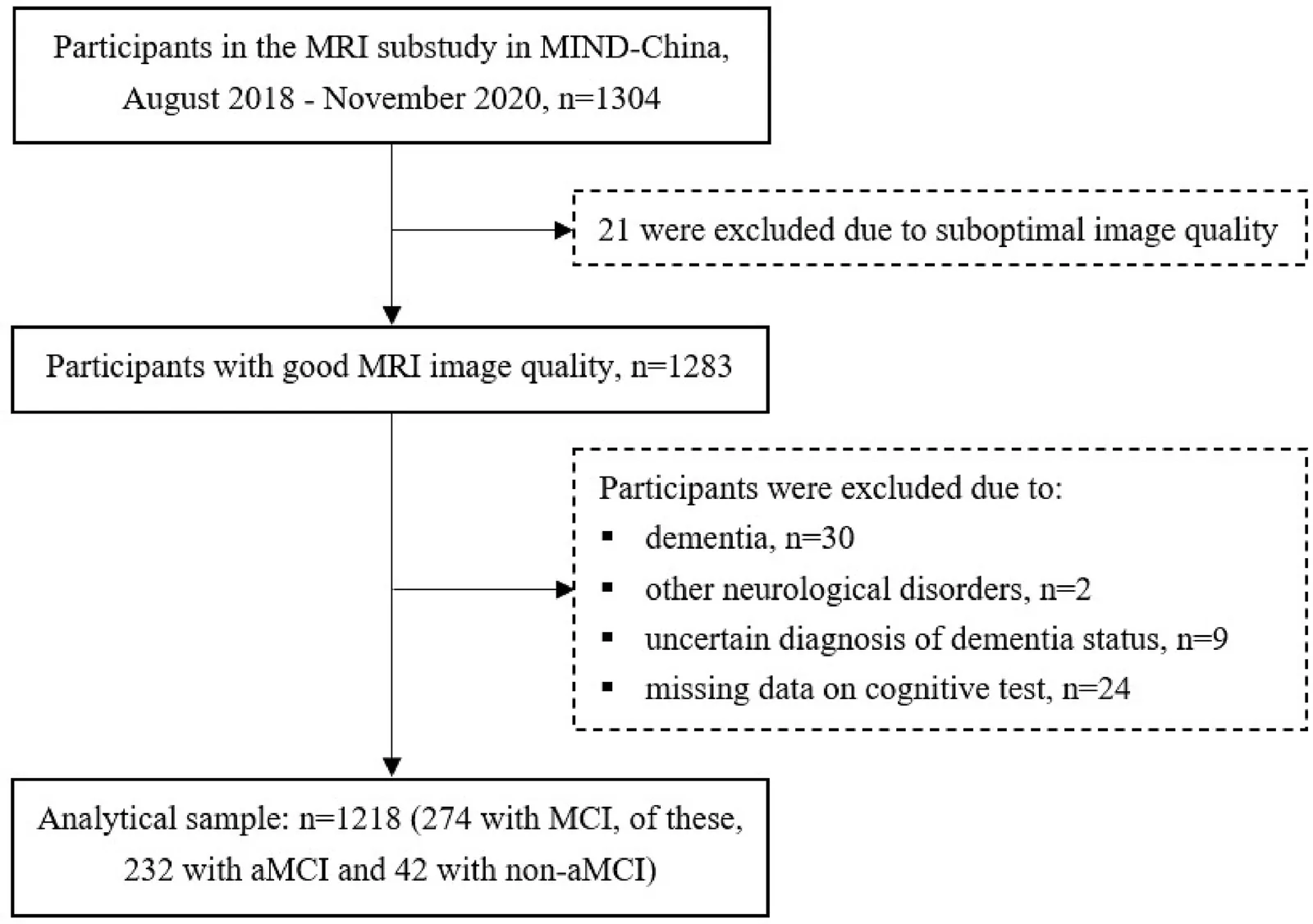
Flowchart of the study participants.

MRI: magnetic resonance imaging; MIND-China: Multimodal Interventions to Delay Dementia and Disability in Rural China; MCI: mild cognitive impairment; aMCI: amnestic mild cognitive impairment; non-aMCI: non-amnestic mild cognitive impairment.

A total of 1304 participants who completed brain magnetic resonance imaging (MRI) scans were included in this population-based cross-sectional study. The study sample was derived from the Multimodal Interventions to Delay Dementia and Disability in Rural China (MIND-China) project,^18,19^ a community-based intervention study targeting residents who were aged ≥60 years and residing in 52 villages of Yanlou Town, Yanggu County, located in western Shandong province. Between March and September 2018, 5765 participants were examined during the baseline examinations. Random selection of 26 villages from all the 52 villages was performed using a cluster-based sampling approach, encompassing a total of 1884 eligible participants, among whom 1304 (69.2%) provided informed consent and eventually underwent the MRI examination between August 2018 and November 2020, as previously reported.^18,20^ The primary reasons that eligible participants who did not undertake brain MRI scanning included contraindications, having a history of major brain disease, or refusal to participate. The mean time interval between cognitive assessment and MRI acquisition was 1.39 years (standard deviation [SD], 0.62). Of the 1304 participants in the initial sample, 86 were excluded from the analysis for the following reasons: suboptimal MRI image quality (n = 21), clinical diagnosis of dementia (n = 30), other neurological conditions (n = 2), unclear dementia status (n = 9), or incomplete data for defining cognitive status (n = 24). A detailed flowchart of study participants is presented in Figure 1.

The study protocol of MIND-China received ethical clearance from the Ethics Committee of Shandong Provincial Hospital in Jinan, China, and MIND-China was registered with the Chinese Clinical Trial Registry (ChiCTR1800017758). The research adhered to the ethical guidelines stated in the Declaration of Helsinki. Written informed consent was obtained from each participant or their legally authorized representative.

### Data collection and definitions

Trained medical personnel conducted face-to-face assessments that included comprehensive interviews, clinical examinations, cognitive testing, and laboratory analyses performed at a local township hospital.^18^ Data collection was conducted following a standardized questionnaire encompassing demographic characteristics (age, sex, and education), metabolic or cardiovascular risk factors (body mass index [BMI], smoking, alcohol consumption, hypertension, diabetes mellitus, and dyslipidemia), and health history (stroke and coronary heart disease).

Educational attainment was assessed based on the total number of years of formal schooling education and subsequently dichotomized into low (no formal schooling) and high levels (any formal schooling). BMI was computed as weight in kilograms divided by height in meters squared (kg/m^2^). Smoking and alcohol consumption status were classified as either “never” or “ever”, according to participants’ self-reported frequency. Hypertension was defined as having a systolic blood pressure of ≥140 mm Hg, a diastolic blood pressure of ≥90 mm Hg, a self-reported history of hypertension, or current use of antihypertensive. Diabetes mellitus was identified based on fasting plasma glucose levels ≥7.0 mmol/L, a self-reported history of clinical diagnosis of diabetes by a physician, or the use of glucose-lowering drugs. Dyslipidemia was defined as having one or more of the following^21^: total cholesterol ≥6.2 mmol/L, triglycerides ≥2.3 mmol/L, low-density lipoprotein cholesterol ≥4.1 mmol/L, high-density lipoprotein cholesterol <1.0 mmol/L, or the use of lipid-lowering agents. Stroke was defined either by the presence of typical neurological deficits or by a self-reported history of stroke. Coronary heart disease, including angina pectoris, myocardial infarction, coronary angioplasty, or coronary artery bypass grafting, was defined based on medical history or evidence from electrocardiography.

### Genotyping of APOE

Peripheral blood samples were obtained in the morning after an overnight fast, and genomic deoxyribonucleic acid (DNA) was subsequently extracted by qualified technicians using the TIANamp Blood DNA Kit (Tiangen Biotech Co., Ltd, Beijing, China). *APOE* genotyping was conducted using multiple-polymerase chain reaction amplification in accordance with standard methods (iGeneTech Bioscience Co., Ltd, Beijing, China). *APOE* genotype was dichotomized into the presence versus absence of the ε4 allele.

### Cognitive assessments and diagnosis of mild cognitive impairment

A comprehensive cognitive battery, as previously described,^19^ was administered to evaluate function of four cognitive domains: episodic memory, verbal fluency, executive function, and attention. In brief, episodic memory was tested with the Auditory Verbal Learning Test, comprising immediate recall, long-delayed free recall, and long-delayed recognition. Verbal fluency was assessed with the Verbal Fluency Test, encompassing animal, fruit, and vegetable categories. Executive function was determined by performance on the backward Digit Span Test and Trail Making Test-B, whereas attention was examined using the forward Digit Span Test and Trail Making Test-A. The raw scores for each of individual neuropsychological tests were converted into z-scores based on the age- and education-specific means and standard deviations derived from all dementia-free participants at baseline examination of MIND-China. The composite domain-specific z-score was calculated by averaging the z-scores of the tests for that specific domain. The composite global cognitive z-score was then calculated as the mean of the composite z-scores from the four cognitive domains.

MCI was defined following the diagnostic framework proposed and operationalized by Petersen and colleagues,^22^ which included: (1) subjective cognitive decline reported by the subject, an informant, or a physician; (2) objective evidence of cognitive impairment in one or more cognitive domains, as determined by standardized cognitive testing; (3) preserved independence in activities of daily living, as measured by the Chinese version of the Activities of Daily Living scale; and (4) absence of dementia, as defined by the Diagnostic and Statistical Manual of Mental Disorders, Fourth Edition criteria.^23^ Subjective cognitive complaints encompass questions concerning difficulties in remembering, forgetting planned activities, and concerns about memory decline. Objective cognitive impairment was defined as a cognitive z-score at least 1.0 SD below the mean of age- and education-matched peers in any of the four cognitive domains.^22,24^ The final diagnosis of MCI was established based on both cognitive test results and consensus among clinical neurologists. Individuals with MCI were further categorized into amnestic MCI (aMCI) when memory impairment was present, and non-amnestic MCI (non-aMCI) when memory function was preserved.

### MRI data acquisition and neuroimaging assessments

As previously reported,^18,25^ structural brain MRI examination was performed for all eligible participants at two sites: Shandong Southwestern Lu Hospital using a Philips Ingenia 3.0 T scanner (n = 1178) and Liaocheng People's Hospital using a Philips Achieva 3.0 T scanner (Philips Healthcare, Best, the Netherlands) (n = 126). The protocol incorporated sagittal 3D T1-weighted fast field echo, axial T2-weighted fast field echo, and sagittal 3D fluid-attenuated inversion recovery (FLAIR) sequences, with scanning parameters consistent with prior publications.^18^ All raw T1-weighted images were visually inspected by two trained researchers who were blinded to clinical data for overt artifacts, including significant head motion (blurring or ghosting), metal artifacts, or incomplete coverage. In cases of ambiguity, a senior neurologist specialized in neuroimaging analysis (L.S.) reviewed the scans to make a final decision. Image processing was conducted using AccuBrain^®^ (BrainNow Medical Technology Ltd, Shenzhen, Guangdong, China), an FDA-approved cloud-based automated platform for segmenting and quantifying global and regional brain volumes.^26^ As described in our previous study,^27^ GM, white matter (WM), and cerebrospinal fluid (CSF) volumes were automatically estimated through T1-weighted images based on similarity measures. TIV was obtained by summing up GM, WM, and CSF volumes, and brain parenchymal volume was calculated as the sum of GM and WM volumes. Specifically, the CSF segmentation protocol included both the internal ventricular system and the extracerebral (subarachnoid) spaces.^28^ For the quantification of white matter hyperintensity (WMH) volume, AccuBrain^®^ employed a series of pre-defined, fixed thresholds for WMH recognition. These thresholds were empirically determined and optimized during the development phase of the algorithm, and were not subject to manual setting or adjustment by the user on a per-scan or per-study basis.^29^ AccuBrain^®^ demonstrates comparable performance with the Lesion Segmentation Tool (LST) in volumetric quantification of WMH under intra-scanner settings, and yields substantially improved inter-scanner reproducibility.^30^ To reduce false positives, the MIND-China team implemented rigorous manual quality-control procedures. All acquired T2-FLAIR images underwent visual inspection to exclude scans with prominent artifacts that may bias intensity-based WMH classification. Following image screening, automated WMH segmentation outputs were further reviewed to identify and exclude systematic errors, such as the misidentification of normal white matter tissue as WMHs.

### Measurements of brain reserve

TIV reaches its maximum size during early adulthood and then remains relatively stable independent of brain aging and neurodegeneration.^31,32^ Therefore, TIV was adopted as an estimate of early-life BR.^33^ Given that BR can change dynamically over the lifespan due to brain aging and cerebral pathology,^7,34^ we used the ratio of total normal brain tissue volume (sum of GM volume and WM volume, excluding WMH volume) to TIV as a proxy for late-life BR.^35^ Indicators of BR in both early- and late-life were standardized to z-scores using their respective means and SDs in subsequent analyses. The z-scores of early- and late-life BR were subsequently categorized into high and low levels based on their median value.

### Statistical analysis

The mean (SD) was presented for continuous variables, and frequencies (%) were reported for categorical variables. Participants’ characteristics by late-life BR levels were compared using the *t*-test for continuous variables and the chi-square test for categorical variables. To assess the linearity of relationships between BR proxies and cognitive z-scores, we employed restricted cubic splines (RCS) with three knots located at the 10th, 50th, and 90th percentiles. The significance of non-linearity was evaluated using likelihood ratio tests, fully adjusted for all covariates. As no significant deviations from linearity were detected (*P*-value for non-linearity >0.05), general linear regression models were used for the analysis. General linear regression analysis was additionally employed to examine the associations of early- and late-life BR with cognitive z-scores across domains of memory, verbal fluency, executive function, attention, and global cognition. To ensure robustness, BR proxies were modeled as both continuous and dichotomous variables. Logistic regression analysis was conducted to examine the relationships between early- and late-life BR proxies and the likelihood of MCI and its subtypes. Additionally, we assessed the joint associations of BR and education with cognitive function by categorizing all participants, according to two dichotomous variables of BR (low and high) and educational attainment (low and high), into low BR and low educational attainment (reference), high BR and low educational attainment, low BR and high educational attainment, and high BR and high educational attainment. Specifically, we first assessed the independent associations of BR and education with cognition in separate models. Subsequently, we included both BR and education as independent predictors within the same multivariable model. This allowed us to assess their joint associations with cognitive scores and MCI. Finally, we introduced an interaction term (to assess synergistic or statistical interaction) between BR and education into the model to investigate whether the association of BR with cognitive outcome was moderated by educational attainment, with a *P*-value for interaction <0.05 indicating the presence of statistical interaction. The stratified analysis between BR and cognitive function by high and low educational attainment was conducted in case of detection of a statistically significant interaction.

The primary results were presented from two models: Model 1 was adjusted for age, sex, *APOE* genotype, and if applicable, for educational level; Model 2 was additionally adjusted for smoking, alcohol consumption, BMI, hypertension, diabetes mellitus, dyslipidemia, stroke, and coronary heart disease because these factors have been associated with brain health and cognitive health.^1,36^ For participants with missing data on specific covariates, including *APOE* genotype, alcohol consumption, and hypertension, a dummy variable was created to represent the missing category. RCS analyses were performed using R version 4.2.3 (R Core Team. The R Foundation for Statistical Computing, Vienna, Austria. https://www.R-project.org/), while all other statistical analyses were conducted with Stata version 16.0 software for Windows (StataCorp, College Station, TX). A two-tailed *P*-value of less than 0.05 was considered statistically significant.

## Results

### Characteristics of the study population

The analytical sample comprised 1218 participants, with a mean age of 69.36 years (SD = 4.25), of these, 58.29% were females, 33.83% had no formal education, and 81.5% were farmers that engaged in manual work on farmland (Table 1). Early-life BR, proxied by TIV, averaged 1409.84 ml (SD = 130.76; range: 932.84–1813.05 ml). Late-life BR, defined as the ratio of normal brain tissue volume-to-TIV, averaged 0.75 (SD = 0.02; range: 0.66–0.83). Compared to the low late-life BR group, participants with high late-life BR were younger and more likely to be female, had lower educational attainment, and had a lower prevalence of hypertension, diabetes, and stroke; higher cognitive z-scores in memory, executive function, attention, and verbal fluency domains and global cognition; and larger brain parenchymal volumes and smaller WMH volumes (all p < 0.05). No significant group differences were observed for smoking, alcohol use, BMI, dyslipidemia, coronary heart disease, *APOE* genotype, or TIV (all p > 0.05, Table 1).

**Table 1.** Characteristics of the study participants in the total sample and by late-life brain reserve levels.

Characteristics	Total sample (n = 1218)	Low late-life BR (n = 609)	High late-life BR (n = 609)	*p*
Age (y), mean (SD)	69.36 (4.25)	70.74 (4.14)	67.99 (3.89)	<0.001
Female sex	710 (58.29)	337 (55.34)	373 (61.25)	0.036
*APOE* ε4 allele*	184 (15.35)	93 (15.55)	91 (15.14)	0.844
Education				0.039
No formal schooling	412 (33.83)	189 (31.03)	223 (36.62)	
Any formal schooling	806 (66.17)	420 (68.97)	386 (63.38)	
Occupation*				0.942
Farmers	981 (80.67)	490 (80.59)	491 (80.76)	
Non-framers	235 (19.33)	118 (19.41)	117 (19.24)	
Cardiovascular factors				
Ever smoking	385 (31.61)	203 (33.33)	182 (29.89)	0.196
Ever alcohol consumption*	471 (38.93)	244 (40.46)	227 (37.40)	0.274
BMI kg/m^2^, mean (SD)	24.98 (3.51)	24.91 (3.57)	25.05 (3.46)	0.469
Hypertension*	816 (67.66)	440 (73.09)	376 (62.25)	<0.001
Diabetes mellitus	185 (15.19)	108 (17.73)	77 (12.64)	0.013
Dyslipidemia	292 (23.97)	149 (24.47)	143 (23.48)	0.687
Health conditions				
Stroke	149 (12.23)	91 (14.94)	58 (9.52)	0.004
Coronary heart disease	217 (17.82)	115 (18.88)	102 (16.75)	0.330
Cognitive z-score, mean (SD)				
Memory	0.13 (0.85)	−0.03 (0.83)	0.29 (0.83)	<0.001
Verbal fluency	0.12 (0.79)	−0.02 (0.78)	0.26 (0.78)	<0.001
Executive function	−0.01 (0.91)	−0.09 (0.84)	0.07 (0.97)	0.002
Attention	0.07 (0.86)	−0.01 (0.83)	0.15 (0.89)	0.001
Global cognition	0.08 (0.65)	−0.04 (0.61)	0.19 (0.67)	<0.001
Brain MRI, mean (SD), ml				
TIV	1409.84 (130.76)	1405.47 (135.81)	1414.22 (125.47)	0.243
Brain parenchymal volume	1060.66 (102.53)	1036.19 (102.27)	1085.12 (96.88)	<0.001
WMH volume	8.48 (10.92)	12.19 (13.33)	4.76 (5.76)	<0.001
MCI	274 (22.50)	169 (27.75)	105 (17.24)	<0.001
aMCI	232 (19.73)	147 (25.04)	85 (14.43)	<0.001
Non-aMCI	42 (4.26)	22 (4.76)	20 (3.82)	0.463

Data were n (%), unless otherwise specified. BR: brain reserve; SD: standard deviation; *APOE*: apolipoprotein E; BMI: body mass index; TIV: total intracranial volume; WMH: white matter hyperintensities; MCI: mild cognitive impairment; aMCI: amnestic mild cognitive impairment; non-aMCI: non-amnestic mild cognitive impairment.

*Data were missing in 19 (1.56%) persons for *APOE* genotype, 2 (0.16%) for occupation, 8 (0.66%) for alcohol consumption, and 12 (0.99%) for hypertension. In subsequent analyses, the missing values were replaced with a dummy variable.

### Associations of early-life and late-life BR with cognitive performance

The RCS analyses indicated linear relationships between both BR measures and cognitive z-scores (all p for nonlinear >0.05; Supplemental Figure 1). After adjusting for age, sex, *APOE* genotype, and education, higher early-life BR (continuous) was significantly associated with higher z-scores in memory and attention (both p < 0.05; Table 2). These significant associations remained after additional adjustment for vascular risk factors. However, early-life BR as a dichotomous variable showed no significant associations. In contrast, higher late-life BR (as a continuous variable) was significantly associated with better performance across all cognitive domains (memory, verbal fluency, executive function, and attention) and global cognition in multivariable-adjusted models (all p < 0.05; Table 2). Consistently, participants in the higher late-life BR category performed significantly better across all cognitive domains than those in the lower BR category (all p < 0.01; Table 2).

**Table 2.** Association of early-life and late-life brain reserve proxies with cognitive performance (n = 1218).

**Brain reserve**	β coefficient (95% confidence interval), cognitive z-score				
proxies	Memory	Verbal fluency	Executive function	Attention	Global cognition
Model 1					
Early-life BR					
Continuous	−0.07 (−0.13, −0.01)*	0.01 (−0.04, 0.07)	0.04 (−0.01, 0.10)	**0.08 (0.02, 0.13)^†^**	0.02 (−0.03, 0.06)
Categorical					
Low	0.00 (reference)	0.00 (reference)	0.00 (reference)	0.00 (reference)	0.00 (reference)
High	−0.06 (−0.18, 0.05)	−0.01 (−0.11, 0.09)	0.05 (−0.06, 0.15)	0.10 (−0.00, 0.20)	0.02 (−0.06, 0.10)
Late-life BR					
Continuous	**0.13 (0.08, 0.18)^‡^**	**0.12** (**0.07, 0.16)^‡^**	**0.06 (0.02, 0.11)^†^**	**0.07 (0.02, 0.12)^†^**	**0.10** (**0.06, 0.13)^‡^**
Categorical					
Low	0.00 (reference)	0.00 (reference)	0.00 (reference)	0.00 (reference)	0.00 (reference)
High	**0.27 (0.17, 0.37) ^‡^**	**0.24** (**0.15, 0.32)^‡^**	**0.15 (0.05, 0.24)^†^**	**0.15 (0.06, 0.24)^†^**	**0.20** (**0.13, 0.27)^‡^**
Model 2					
Early-life BR					
Continuous	**−0.08 (−0.14, −0.02)^†^**	0.00 (−0.05, 0.06)	0.03 (−0.02, 0.09)	0.07 (0.02, 0.12)*	0.01 (−0.03, 0.05)
Categorical					
Low	0.00 (reference)	0.00 (reference)	0.00 (reference)	0.00 (reference)	0.00 (reference)
High	−0.07 (−0.18, 0.05)	−0.02 (−0.12, 0.08)	0.03 (−0.07, 0.14)	0.09 (−0.01, 0.19)	0.01 (−0.07, 0.09)
Late-life BR					
Continuous	**0.13 (0.08, 0.18)^‡^**	**0.11** (**0.06, 0.15)^‡^**	0.06 (0.01, 0.10)*	**0.06 (0.02, 0.11)^†^**	**0.09** (**0.05, 0.12)^‡^**
Categorical					
Low	0.00 (reference)	0.00 (reference)	0.00 (reference)	0.00 (reference)	0.00 (reference)
High	**0.27 (0.17, 0.36) ^‡^**	**0.22** (**0.13, 0.31)^‡^**	**0.13 (0.04, 0.22)^†^**	**0.14 (0.05, 0.23)^†^**	**0.19** (**0.12, 0.26)^‡^**

Model 1 was controlled for age, sex, *APOE* genotype, and education. Model 2 was additionally adjusted for smoking, alcohol consumption, body mass index, hypertension, diabetes mellitus, hyperlipidemia, stroke, and coronary heart disease. BR: brain reserve. *p < 0.05, ^†^*p* < 0.01, ^‡^*p* < 0.001.

### Associations of early-life and late-life BR with MCI and its subtypes

As a continuous variable, higher early-life BR was significantly associated with an approximately 35% lower odds of non-aMCI in fully adjusted models (p < 0.05, Table 3), but not with MCI or aMCI. As a dichotomous variable, early-life BR had no significant associations with MCI or its subtypes (p > 0.05). In contrast, higher late-life BR, both as a continuous and dichotomous variables, was consistently associated with lower likelihoods of both MCI and aMCI. Specifically, as a continuous variable, late-life BR was significantly associated with approximately 25% reduced likelihoods of MCI and aMCI, and as a dichotomous variable, higher late-life BR was significantly associated with approximately 47% and 52% lower odds of MCI and aMCI, respectively, in fully adjusted models (all p < 0.001, Table 3).

**Table 3.** Association of early-life and late-life brain reserve proxies with mild cognitive impairment and subtypes (n = 1218).

Brain reserve	Odd ratio (95% confidence interval)^a^		
proxies	MCI (n = 274)	aMCI (n = 232)	non-aMCI (n = 42)
Model 1			
Early-life BR			
Continuous	0.92 (0.77, 1.10)	0.98 (0.81, 1.19)	0.63 (0.42, 0.95)*
Categorical			
Low	1.00 (reference)	1.00 (reference)	1.00 (reference)
High	0 .93 (0.67, 1.29)	1.02 (0.72, 1.45)	0.54 (0.25, 1.18)
Late-life BR			
Continuous	**0.75 (0.65, 0.87)^‡^**	**0.74** (**0.64, 0.87)^‡^**	0.82 (0.58, 1.14)
Categorical			
Low	1.00 (reference)	1.00 (reference)	1.00 (reference)
High	**0.53 (0.39, 0.71)^‡^**	**0.49** (**0.36, 0.68)^‡^**	0.80 (0.41, 1.56)
Model 2			
Early-life BR			
Continuous	0.94 (0.78, 1.12)	1.01 (0.83, 1.22)	0.65 (0.43, 0.99)*
Categorical			
Low	1.00 (reference)	1.00 (reference)	1.00 (reference)
High	0 .95 (0.68, 1.32)	1.04 (0.73, 1.48)	0.60 (0.26, 1.37)
Late-life BR			
Continuous	**0.75 (0.65, 0.88)^‡^**	**0.74** (**0.63, 0.86)^‡^**	0.90 (0.62, 1.28)
Categorical			
Low	1.00 (reference)	1.00 (reference)	1.00 (reference)
High	**0.53 (0.39, 0.72)^‡^**	**0.48** (**0.35, 0.67)^‡^**	0.94 (0.47, 1.89)

^a^ The odds ratios (95% confidence intervals) of MCI were derived from binary logistic regression models and the odds ratios (95% confidence intervals) of aMCI and non-aMCI were derived from multinomial logistic regression models, in which individuals without MCI (n = 944) were used as reference group in the modelling.

Model 1 was controlled for age, sex, *APOE* genotype, and education. Model 2 was additionally adjusted for smoking, alcohol consumption, body mass index, hypertension, diabetes mellitus, hyperlipidemia, stroke, and coronary heart disease. BR: brain reserve; MCI: mild cognitive impairment; aMCI: amnestic mild cognitive impairment; non-aMCI: non-amnestic mild cognitive impairment.

*p < 0.05, ^‡^*p* < 0.001.

### Associations between BR-education joint exposures and cognitive performance

**Figure 2.**
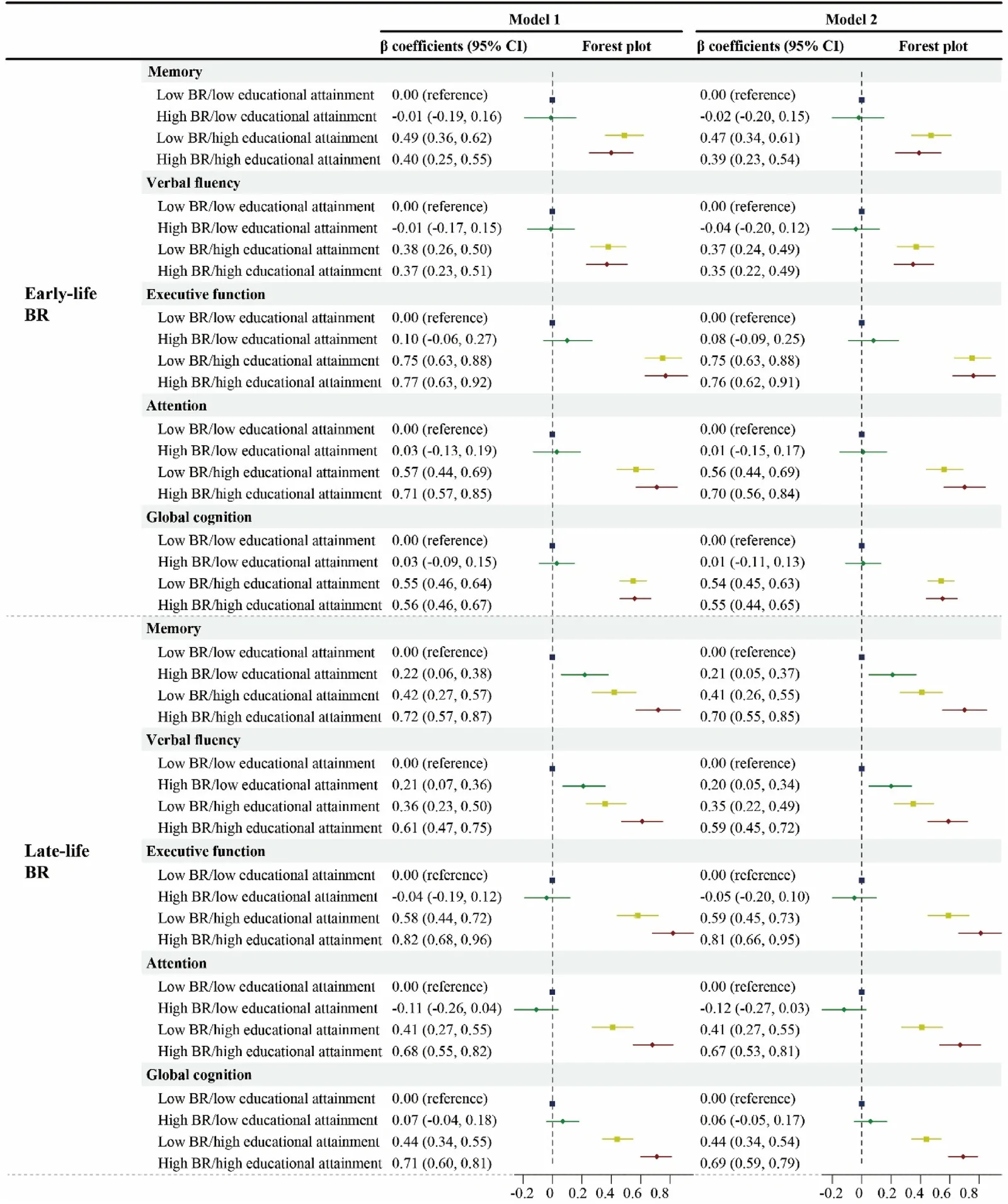
Association of joint brain reserve at early- and late-life and educational attainment with cognitive performance. Data were β coefficients (95% confidence intervals) of cognitive z-score associated with joint exposures to early- or late-life BR and educational attainment, derived from different models. Model 1 was controlled for age, sex, and *APOE* genotype. Model 2 was additionally adjusted for smoking, alcohol consumption, body mass index, hypertension, diabetes mellitus, hyperlipidemia, stroke, and coronary heart disease. CI: confidence interval; BR: brain reserve.

The associations of BR and educational attainment with cognitive performance differed depending on life stage when BR was assessed. In models of early-life BR, cognitive performance did not differ by BR level among individuals with low educational attainment. However, participants with high educational attainment demonstrated consistently superior cognitive performance across all domains and global cognition, independent of their early-life BR status (all p < 0.001; Figure 2). In contrast, for late-life BR, both BR level and educational attainment were associated with cognitive outcomes in a dose-response manner. Specifically, for memory and verbal fluency, a significant linear trend was evident across the four BR/education joint-exposure groups (Figure 2). In fully adjusted models using the low BR/low educational attainment group as reference, the high BR/high educational attainment combination showed the strongest positive associations with z-scores of memory (β = 0.70, 95% CI: 0.55–0.85) and verbal fluency (β = 0.59, 95% CI: 0.45–0.72), followed by the low BR/high educational attainment group (memory: β = 0.41, 95% CI: 0.26–0.55; verbal fluency: β = 0.35, 95% CI: 0.22–0.49), with the high BR/low educational attainment joint group showing more modest associations with memory (β = 0.21, 95% CI: 0.05–0.37) and verbal fluency (β = 0.20, 95% CI: 0.05–0.34) (Figure 2).

### Associations between BR-education joint exposures and MCI and its subtypes

**Figure 3.**
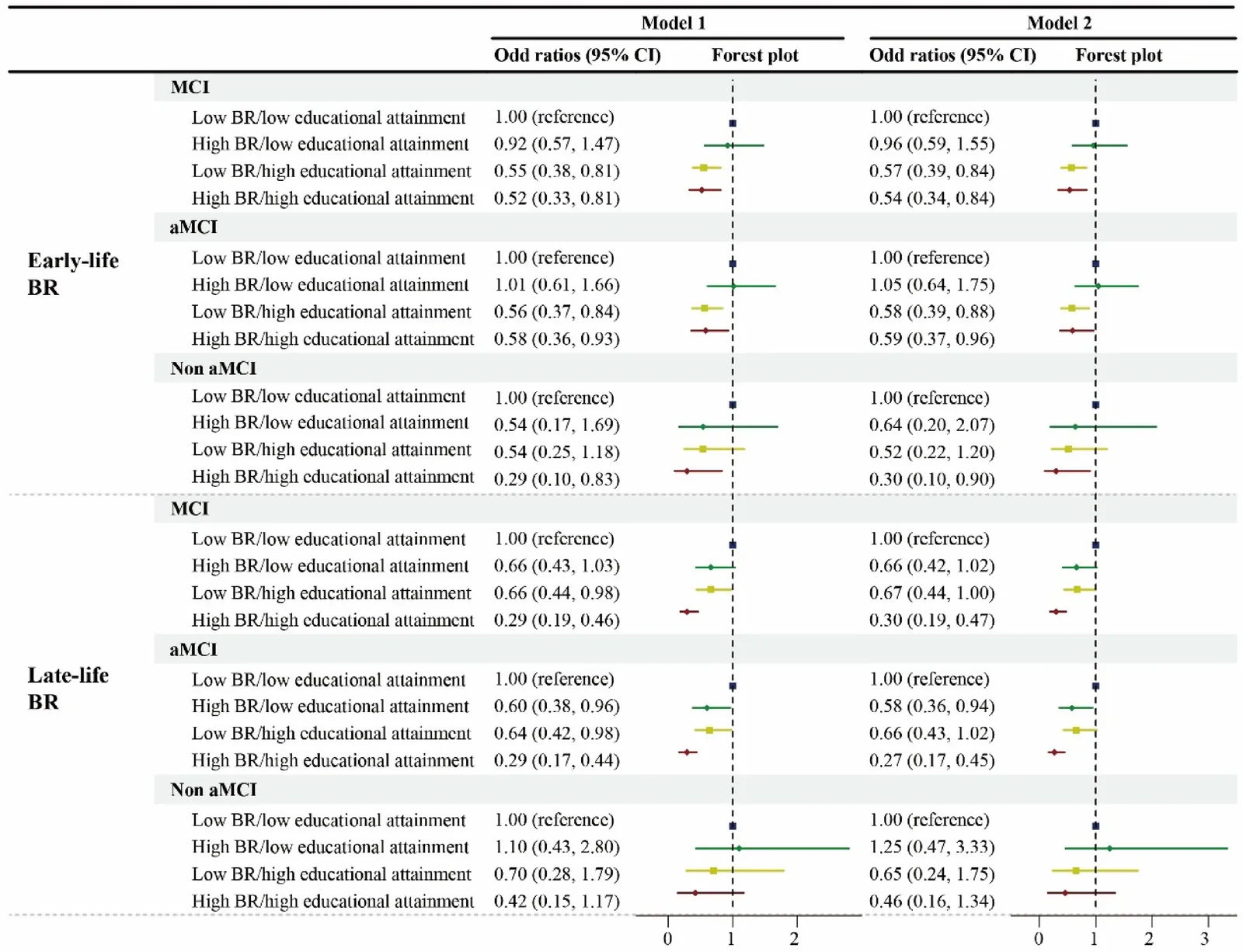
Association of joint brain reserve at early- and late-life and educational attainment with MCI and its subtypes. Data were odds ratios (95% confidence intervals) of MCI or subtypes of MCI associated with joint exposures to early- or late-life BR and educational attainment, derived from different models. Model 1 was controlled for age, sex, and *APOE* genotype. Model 2 was additionally adjusted for smoking, alcohol consumption, body mass index, hypertension, diabetes mellitus, hyperlipidemia, stroke, and coronary heart disease. CI: confidence interval; BR: brain reserve; MCI: mild cognitive impairment; aMCI: amnestic mild cognitive impairment; non-aMCI: non-amnestic mild cognitive impairment.

The associations of joint exposures to BR and educational attainment with MCI and its subtypes demonstrated distinct patterns depending on the life stage at which BR was assessed. For early-life BR, relative to the low BR/low educational attainment group, individuals with high BR/low educational attainment exhibited no significant associations with MCI and its subtypes. In contrast, individuals with low BR/high educational attainment had lower odds of MCI (OR = 0.57) and aMCI (OR = 0.58), and those with high BR/high educational attainment also exhibited reduced odds of MCI (OR = 0.54), aMCI (OR = 0.59), and non-aMCI (OR = 0.30). For late-life BR, compared to the group with low BR/low educational attainment, significant reductions in odds of MCI were found in those with high BR/low educational attainment (OR = 0.58) and low BR/high educational attainment (OR = 0.67), with the most pronounced associations seen in the high BR/high educational attainment group for both MCI (OR = 0.30) and aMCI (OR = 0.27). The joint exposures between early- and late-life BR and educational attainment showed no significant associations with non-aMCI (p > 0.05). All reported estimates were derived from fully adjusted models (Figure 3).

### Interactions between BR and educational attainment on cognitive outcomes

**Figure 4.**
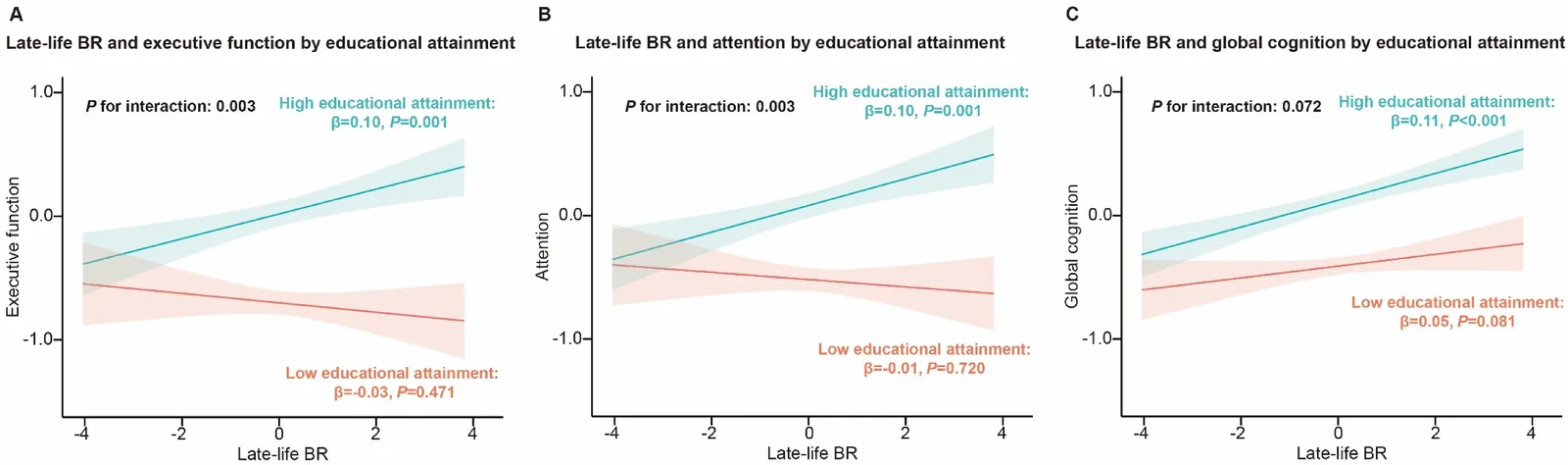
Interaction of educational attainment and late-life brain reserve proxy on cognitive performance. Plots illustrate the significant moderating role of educational attainment in the associations between late-life BR and (A) executive function, (B) attention, and (C) global cognition. Educational attainment was dichotomized as high (any formal school education; green dotted line) or low (no formal school education; red dotted line). Shaded areas denote 95% confidence intervals. Analyses were adjusted for age, sex, *APOE* genotype, smoking, alcohol consumption, body mass index, hypertension, diabetes mellitus, hyperlipidemia, stroke, and coronary heart disease. BR: brain reserve; *APOE*: apolipoprotein E gene.

Statistically significant interactions were observed between late-life BR and educational attainment on executive function (p for interaction = 0.003) and attention (p for interaction = 0.003), with a marginal interaction on global cognition (p for interaction = 0.072). Stratified analyses by educational attainment demonstrated that among participants with high educational attainment, higher late-life BR was significantly associated with better performance in executive function, attention, and global cognition, whereas no significant associations were found with any of the examined cognitive z-scores among those with low educational attainment (Figure 4). Additionally, no significant interactions between late-life BR and educational attainment were observed for memory, verbal fluency, MCI, or its subtypes (p for all interactions >0.05). Similarly, early-life BR showed no significant interactions with educational attainment on any of the examined cognitive outcomes (p for all interactions >0.05).

### Sensitivity analysis

To assess the robustness of our primary findings to potential site-related heterogeneity in the multicenter imaging data, we conducted a sensitivity analysis. Specifically, all imaging data were reprocessed using the ComBat harmonization method to correct for batch effects across acquisition sites.^37^ Subsequently, all statistical analyses were repeated using the harmonized data. Detailed results of this sensitivity analysis are provided in the Supplemental Material (Supplemental Tables 1–3 and Supplemental Figures 2–5). Overall, the results were largely consistent with those of the original analysis, supporting the robustness of our main conclusions.

## Discussion

In this population-based cross-sectional study of dementia-free older adults in rural China, the key findings were summarized as follows: (1) higher late-life BR, but not early-life BR, was independently associated with superior performance in memory, verbal fluency, executive function, attention, and global cognition, alongside a lower likelihood of MCI and aMCI; (2) higher late-life BR was linked to better cognitive performance in memory and verbal fluency, as well as a reduced likelihood of aMCI, even among people with no formal schooling; and (3) among people with any formal schooling, higher late-life BR was related to better performance in executive function, attention, and global cognition, whereas no such associations were observed in those with no formal schooling. These findings highlight the potential role of late-life BR, particularly in the context of educational attainment, in preserving cognitive function and reducing the risk of cognitive impairment.

In our study sample, people with high late-life BR were younger and more likely to be females, and had lower educational attainment and a lower prevalence of vascular/metabolic conditions than those with low late-life BR. This profile may reflect the fact that younger age was associated with fewer age-related brain injuries, aligning with higher BR; that females had a lower cerebrovascular lesion burden (e.g., stroke and WMH) than males, thus having high BR; and that females had significantly lower education, thus having an overall lower educational level in the high BR group. Our analysis revealed that elevated late-life BR, rather than early-life BR, was consistently associated with better performance across all the assessed cognitive domains, aligning with findings from the prospective SMART-MR study.^38^ A clinic-based retrospective case-control study showed that a larger TIV could offer greater reserve capacity against Alzheimer's disease.^39^ Additionally, a study involving cognitively and ethnically diverse older adults found that a greater TIV was correlated with enhanced performance in semantic memory, executive function, and spatial abilities, after adjusting for age, sex, and educational attainment.^40^ However, our population-based study did not find evidence that TIV, serving as a surrogate measure of early-life BR, was linked to cognitive performance after adjustments for demographic characteristics, lifestyle behaviors, and cardiovascular and metabolic risk factors. This suggests that vascular health may substantially influence the relationship between early-life BR and late-life cognitive performance, potentially offsetting its cognitive benefits in later life.^41^ In contrast, late-life BR, indicative of the preserved volume of healthy brain tissue, showed a significant association with cognitive performance. Given the accumulation of vascular risk factors with aging and their close relationship with structural brain injury (e.g., increased WMH and reduced brain tissue volume),^42,43^ late-life BR may exert a more pronounced influence on cognitive function compared with early-life BR. This is particularly evident when cardiovascular risk factors are taken into consideration.

Data from the Amsterdam Dementia Cohort did not support the association between TIV and dementia or cognitive decline.^44^ Similarly, neither TIV nor head circumstance was linked to cognitive change in a study of rural older adults in South Korea.^45^ The US Atherosclerosis Risk in Communities study of middle-aged people found that individuals with MCI showed volumetric loss in cortical lobes, particularly in the temporal lobe.^46^ Additionally, our previous study linked global WMH burden with MCI,^47^ emphasizing the importance of considering pathological brain regions in relation to cognitive function. Together, these findings indicate that the ratio of healthy brain tissue to TIV, rather than TIV *per se*, is more sensitive in capturing the association of late-life BR with cognitive health.

An important finding of our study was that educational attainment could modify the association between late-life BR and cognitive profile. Notably, a greater late-life BR was linked to superior performance in executive function, attention, and global cognition among participants with any formal schooling, whereas no such associations were observed in those with no formal schooling. This finding aligns with a study from the UC Davis Diversity Cohort, which reported that the relationship between brain atrophy and global cognitive decline was more evident in individuals with higher educational attainment (≥16 years) than in those with lower education.^48^ Educational attainment may facilitate functional compensation, a key component of cognitive reserve, by enabling the brain to recruit alternative neural networks to maintain performance even as structural integrity declines.^49^ Our study extends this moderating effect not only on global cognition but also on specific cognitive domains of executive function and attention. Furthermore, our study demonstrated that the moderating role of education persisted even in populations with limited access to compulsory education, rather than being confined to well-educated cohorts. The interaction between late-life BR and educational attainment highlights the potential that promoting healthy brain aging depends on optimizing lifelong factors, such as minimizing brain injury and maximizing educational opportunities. In addition to compensatory mechanism, higher educational attainment may enhance the benefits from structural brain integrity in preserving cognitive function.

The synergistic associations of BR and educational attainment with cognitive outcomes observed in our study are consistent with the existing literature.^50,51^ Moreover, our results indicate that late-life BR, rather than early-life BR, is positively associated with overall cognitive function, including memory and language abilities, even among individuals with low educational attainment. Specific brain regions, such as the hippocampus and frontal lobe, are crucial to memory and language functions. Higher volumes in these brain areas independently contribute to domain-specific resilience. For example, greater hippocampal volume is strongly correlated with memory performance, regardless of educational attainment.^52^ These findings suggest that both educational attainment and BR are crucial for late-life cognitive health, with their influences being likely to interwoven throughout the lifespan. Taken together, the relationship between reserve and cognitive profile is complex, with educational attainment and BR both playing important roles in maintaining cognitive function throughout the lifespan.

TIV, a fixed brain volume that reaches its maximum in adolescence, may reflect early-life brain development more accurately than it reflects an individual's available BR at the time of cognitive assessment. As expected, education was positively associated with TIV, implying that cognitive stimulation in early life may partly facilitate structural brain development. Additionally, we found that older age was associated with lower BR in both early- and late-life, highlighting the dynamic nature of BR as people age. Moreover, hypertension, diabetes mellitus, and stroke, which are risk factors for cerebrovascular disorders, were associated with decreased late-life BR. Therefore, white matter lesions were excluded when assessing late-life BR. The amount of undamaged brain tissue may serve as a more appropriate and precise proxy for BR when exploring cognitive profile in old age.

Our large-scale population-based study underscored the robust associations between late-life BR and cognitive function. These findings extend previous reports that are largely from highly educated urban populations in high-income Western societies to the understudied rural populations with limited education, revealing that even primary education remains a crucial modifier. Finally, our rigorous neuroimaging protocol and standardized TIV estimates are crucial for the reliable assessment of BR proxy. Nonetheless, several limitations of our study should be discussed. First, the cross-sectional design precludes causal inference regarding the relationships between BR and cognitive outcomes, and the observed cross-sectional associations could be influenced by selective survival bias. Second, the exploratory nature of our work reflects the current lack of standardized definitions and internationally accepted proxies for early- and late-life BR. In addition, educational attainment as a proxy for CR may not capture the full complexity of lifelong intellectual and social engagement. Our study engaged an understudied rural older Chinese population. While this was a strength of our study, the findings may not be fully generalizable to urban populations or other ethnic groups with different educational systems. Finally, the lack of data on reliable Alzheimer's disease biomarkers, such as CSF or amyloid-beta PET-CT biomarkers, may have influenced the precision interpretation of late-life BR estimates.

In summary, this population-based cross-sectional study observed that greater late-life BR was associated with superior cognitive performance and a reduced likelihood of MCI and aMCI. Furthermore, the association between greater late-life BR and better cognitive performance was evident even in individuals with no formal schooling. Finally, formal school education may enhance the association between late-life BR and cognition. These findings suggest that maintaining late-life BR and improving educational attainment may be effective for promoting cognitive health in aging populations. Because MIND-China is an ongoing multimodal interventional study, with promotion of active lifestyles and CR (e.g., physical exercise, social activity, and cognitive training) being the main components of the non-pharmacologic interventions, future studies should seek to clarify whether these interventional approaches could help maintain late-life BR, and thus, benefit cognitive health and delay the onset of the dementia syndrome.

## Supplemental Material

sj-docx-1-alz-10.1177_13872877261469556 - Supplemental material for Early- and late-life brain reserve proxies and their interactions with education on cognitive outcomes in older adults: A population-based MRI study Supplemental material, sj-docx-1-alz-10.1177_13872877261469556 for Early- and late-life brain reserve proxies and their interactions with education on cognitive outcomes in older adults: A population-based MRI study by Xiaojuan Han, Yuanjing Li, Jiafeng Wang, Xinyu Liu, Enming Cao, Yiming Song, Lin Song, Lin Cong, Qinghua Zhang, Shi Tang, Yongxiang Wang, Yifeng Du and Chengxuan Qiu in Journal of Alzheimer's Disease

## References

[1] Livingston G Huntley J Liu KY , et al. Dementia prevention, intervention, and care: 2024 report of the Lancet standing Commission. Lancet 2024; 404: 572–628. 39096926 10.1016/S0140-6736(24)01296-0

[2] Kivipelto M Mangialasche F Snyder HM , et al. World-Wide FINGERS Network: a global approach to risk reduction and prevention of dementia. Alzheimers Dement 2020; 16: 1078–1094. 32627328 10.1002/alz.12123 PMC9527644

[3] Baker LD Espeland MA Whitmer RA , et al. Structured vs self-guided multidomain lifestyle interventions for global cognitive function: the US POINTER randomized clinical trial. JAMA 2025; 334: 681–691. 40720610 10.1001/jama.2025.12923 PMC12305445

[4] Lebel C Beaulieu C . Longitudinal development of human brain wiring continues from childhood into adulthood. J Neurosci 2011; 31: 10937–10947. 21795544 10.1523/JNEUROSCI.5302-10.2011 PMC6623097

[5] Perneczky R Wagenpfeil S Lunetta KL , et al. Head circumference, atrophy, and cognition: implications for brain reserve in Alzheimer disease. Neurology 2010; 75: 137–142. 20625166 10.1212/WNL.0b013e3181e7ca97 PMC2905931

[6] van Loenhoud AC Groot C Vogel JW , et al. Is intracranial volume a suitable proxy for brain reserve? Alzheimers Res Ther 2018; 10: 91. 30205838 10.1186/s13195-018-0408-5 PMC6134772

[7] Stern Y . An approach to studying the neural correlates of reserve. Brain Imaging Behav 2017; 11: 410–416. 27450378 10.1007/s11682-016-9566-x PMC5810375

[8] Yang K Chen G Sheng C , et al. Cognitive reserve, brain reserve, APOE-epsilon4, and cognition in individuals with subjective cognitive decline in the SILCODE study. J Alzheimers Dis 2020; 76: 249–260. 32444543 10.3233/JAD-200082

[9] Stern Y Arenaza-Urquijo EM Bartres-Faz D , et al. Whitepaper: defining and investigating cognitive reserve, brain reserve, and brain maintenance. Alzheimers Dement 2020; 16: 1305–1311. 30222945 10.1016/j.jalz.2018.07.219 PMC6417987

[10] Stern Y Barnes CA Grady C , et al. Brain reserve, cognitive reserve, compensation, and maintenance: operationalization, validity, and mechanisms of cognitive resilience. Neurobiol Aging 2019; 83: 124–129. 31732015 10.1016/j.neurobiolaging.2019.03.022 PMC6859943

[11] Lovden M Fratiglioni L Glymour MM , et al. Education and cognitive functioning across the life span. Psychol Sci Public Interest 2020; 21: 6–41. 32772803 10.1177/1529100620920576 PMC7425377

[12] Le Carret N Lafont S Mayo W , et al. The effect of education on cognitive performances and its implication for the constitution of the cognitive reserve. Dev Neuropsychol 2003; 23: 317–337. 12740188 10.1207/S15326942DN2303_1

[13] Han X Li Y Wang J , et al. Associations between lifelong cognitive reserve, Alzheimer’s disease-related plasma biomarkers, and cognitive function in dementia-free older adults: a population-based study. J Alzheimers Dis 2025; 103: 821–832. 39791378 10.1177/13872877241306448

[14] Amieva H Mokri H Le Goff M , et al. Compensatory mechanisms in higher-educated subjects with Alzheimer’s disease: a study of 20 years of cognitive decline. Brain 2014; 137: 1167–1175. 24578544 10.1093/brain/awu035

[15] Bai W Chen P Cai H , et al. Worldwide prevalence of mild cognitive impairment among community dwellers aged 50 years and older: a meta-analysis and systematic review of epidemiology studies. Age Ageing 2022; 51: afac173. 10.1093/ageing/afac173 35977150

[16] Arenaza-Urquijo EM Landeau B La Joie R , et al. Relationships between years of education and gray matter volume, metabolism and functional connectivity in healthy elders. Neuroimage 2013; 83: 450–457. 23796547 10.1016/j.neuroimage.2013.06.053

[17] Liu Y Julkunen V Paajanen T , et al. Education increases reserve against Alzheimer’s disease–evidence from structural MRI analysis. Neuroradiology 2012; 54: 929–938. 22246242 10.1007/s00234-012-1005-0 PMC3435513

[18] Wang Y Han X Zhang X , et al. Health status and risk profiles for brain aging of rural-dwelling older adults: data from the interdisciplinary baseline assessments in MIND-China. Alzheimers Dement (N Y) 2022; 8: e12254. 10.1002/trc2.12254 PMC9009233 35441085

[19] Cong L Ren Y Wang Y , et al. Mild cognitive impairment among rural-dwelling older adults in China: a community-based study. Alzheimers Dement 2023; 19: 56–66. 35262288 10.1002/alz.12629 PMC10078715

[20] Wang J Han X Li Y , et al. Strategic lacunes associated with mild cognitive impairment in rural Chinese older adults: a population-based study. Stroke 2024; 55: 1288–1298. 38511349 10.1161/STROKEAHA.123.044469

[21] Joint committee for guideline revision. 2016 Chinese guidelines for the management of dyslipidemia in adults. J Geriatr Cardiol 2018; 15: 1–29. 29434622 10.11909/j.issn.1671-5411.2018.01.011 PMC5803534

[22] Petersen RC Roberts RO Knopman DS , et al. Prevalence of mild cognitive impairment is higher in men. The Mayo Clinic Study of Aging. Neurology 2010; 75: 889–897. 20820000 10.1212/WNL.0b013e3181f11d85 PMC2938972

[23] American Psychiatric Association. Diagnostic and statistical manual of mental disorders (4th ed., text rev.). American Psychiatric Association, 2000.

[24] Di Carlo A Lamassa M Baldereschi M , et al. CIND And MCI in the Italian elderly: frequency, vascular risk factors, progression to dementia. Neurology 2007; 68: 1909–1916. 17536047 10.1212/01.wnl.0000263132.99055.0d

[25] Song L Han X Li Y , et al. Thalamic gray matter volume mediates the association between KIBRA polymorphism and olfactory function among older adults: a population-based study. Cereb Cortex 2023; 33: 3664–3673. 35972417 10.1093/cercor/bhac299 PMC10068283

[26] Zhao L Luo Y Mok V , et al. Automated brain volumetric measures with AccuBrain: version comparison in accuracy, reproducibility and application for diagnosis. BMC Med Imaging 2022; 22: 117. 35787256 10.1186/s12880-022-00841-2 PMC9252062

[27] Wang M Li Y Cong L , et al. High-density lipoprotein cholesterol and brain aging amongst rural-dwelling older adults: a population-based magnetic resonance imaging study. Eur J Neurol 2021; 28: 2882–2892. 34031948 10.1111/ene.14939

[28] Malone IB Leung KK Clegg S , et al. Accurate automatic estimation of total intracranial volume: a nuisance variable with less nuisance. Neuroimage 2015; 104: 366–372. 25255942 10.1016/j.neuroimage.2014.09.034 PMC4265726

[29] Shi L Wang D Liu S , et al. Automated quantification of white matter lesion in magnetic resonance imaging of patients with acute infarction. J Neurosci Methods 2013; 213: 138–146. 23261771 10.1016/j.jneumeth.2012.12.014

[30] Guo C Niu K Luo Y , et al. Intra-scanner and inter-scanner reproducibility of automatic white matter hyperintensities quantification. Front Neurosci 2019; 13: 679. 31354406 10.3389/fnins.2019.00679 PMC6635556

[31] Lenroot RK Giedd JN . Brain development in children and adolescents: insights from anatomical magnetic resonance imaging. Neurosci Biobehav Rev 2006; 30: 718–729. 16887188 10.1016/j.neubiorev.2006.06.001

[32] Courchesne E Chisum HJ Townsend J , et al. Normal brain development and aging: quantitative analysis at in vivo MR imaging in healthy volunteers. Radiology 2000; 216: 672–682. 10966694 10.1148/radiology.216.3.r00au37672

[33] Yoon HJ Kim SG Kim SH , et al. Associations between brain reserve proxies and clinical progression in Alzheimer’s disease dementia. Int J Environ Res Public Health 2021; 18: 12159. 34831913 10.3390/ijerph182212159 PMC8625916

[34] Almeida RP Schultz SA Austin BP , et al. Effect of cognitive reserve on age-related changes in cerebrospinal fluid biomarkers of Alzheimer disease. JAMA Neurol 2015; 72: 699–706. 25893879 10.1001/jamaneurol.2015.0098 PMC4639566

[35] Zijlmans JL Vernooij MW Ikram MA , et al. The role of cognitive and brain reserve in late-life depressive events: the Rotterdam Study. J Affect Disord 2023; 320: 211–217. 36183828 10.1016/j.jad.2022.09.145

[36] Qiu C Fratiglioni L . A major role for cardiovascular burden in age-related cognitive decline. Nat Rev Cardiol 2015; 12: 267–277. 25583619 10.1038/nrcardio.2014.223

[37] Johnson WE Li C Rabinovic A . Adjusting batch effects in microarray expression data using empirical Bayes methods. Biostatistics 2007; 8: 118–127. 16632515 10.1093/biostatistics/kxj037

[38] Vonk JMJ Ghaznawi R Zwartbol MHT , et al. The role of cognitive and brain reserve in memory decline and atrophy rate in mid and late-life: the SMART-MR study. Cortex 2022; 148: 204–214. 35189525 10.1016/j.cortex.2021.11.022 PMC11018269

[39] Jenkins R Fox NC Rossor AM , et al. Intracranial volume and Alzheimer disease: evidence against the cerebral reserve hypothesis. Arch Neurol 2000; 57: 220–224. 10681081 10.1001/archneur.57.2.220

[40] Farias ST Mungas D Reed B , et al. Maximal brain size remains an important predictor of cognition in old age, independent of current brain pathology. Neurobiol Aging 2012; 33: 1758–1768. 21531482 10.1016/j.neurobiolaging.2011.03.017 PMC3177982

[41] Zhou H Lu WJ Zhang ZJ , et al. [Study of cognitive function and brain volume in type 2 diabetic patients]. Zhonghua Yi Xue Za Zhi 2010; 90: 327–331. 20368055

[42] Dickie DA Ritchie SJ Cox SR , et al. Vascular risk factors and progression of white matter hyperintensities in the Lothian Birth Cohort 1936. Neurobiol Aging 2016; 42: 116–123. 27143428 10.1016/j.neurobiolaging.2016.03.011 PMC4869590

[43] Lane CA Barnes J Nicholas JM , et al. Associations between vascular risk across adulthood and brain pathology in late life: evidence from a British Birth Cohort. JAMA Neurol 2020; 77: 175–183. 31682678 10.1001/jamaneurol.2019.3774 PMC6830432

[44] van Loenhoud AC Groot C Bocancea DI , et al. Association of education and intracranial volume with cognitive trajectories and mortality rates across the Alzheimer disease continuum. Neurology 2022; 98: e1679–e1691. 10.1212/WNL.0000000000200116 PMC9052567 35314498

[45] Kwon OD Choi SY Bae J . Association of head circumference with cognitive decline and symptoms of depression in elderly: a 3-year prospective study. Yeungnam Univ J Med 2018; 35: 205–212. 31620595 10.12701/yujm.2018.35.2.205 PMC6784694

[46] Mijares Diaz F Orlando A Schneider AL , et al. Differences in brain volume in individuals with mild cognitive impairment and normal cognition across different anatomical regions: the Atherosclerosis Risk in Communities (ARIC) study. J Alzheimers Dis 2025; 104: 43–51. 39865685 10.1177/13872877251313816

[47] Li Y Cong L Hou T , et al. Characterizing global and regional brain structures in amnestic mild cognitive impairment among rural residents: a population-based study. J Alzheimers Dis 2021; 80: 1429–1438. 33682713 10.3233/JAD-201372

[48] Mungas D Gavett B Fletcher E , et al. Education amplifies brain atrophy effect on cognitive decline: implications for cognitive reserve. Neurobiol Aging 2018; 68: 142–150. 29798764 10.1016/j.neurobiolaging.2018.04.002 PMC5993638

[49] Cabeza R Albert M Belleville S , et al. Maintenance, reserve and compensation: the cognitive neuroscience of healthy ageing. Nat Rev Neurosci 2018; 19: 701–710. 30305711 10.1038/s41583-018-0068-2 PMC6472256

[50] Lamballais S Zijlmans JL Vernooij MW , et al. The risk of dementia in relation to cognitive and brain reserve. J Alzheimers Dis 2020; 77: 607–618. 32741820 10.3233/JAD-200264 PMC7592692

[51] Groot C van Loenhoud AC Barkhof F , et al. Differential effects of cognitive reserve and brain reserve on cognition in Alzheimer disease. Neurology 2018; 90: e149–e156. 10.1212/WNL.0000000000004802 29237798

[52] Lovden M Pagin A Bartres-Faz D , et al. No moderating influence of education on the association between changes in hippocampus volume and memory performance in aging. Aging Brain 2023; 4: 100082. 37457634 10.1016/j.nbas.2023.100082 PMC10338350

