# Supplementary material for: Early- and late-life brain reserve proxies and their interactions with education on cognitive outcomes in older adults: A population-based MRI study
Source: J Alzheimers Dis. 2026 Jul 20;113(1):450–63. doi: 10.1177/13872877261469556 (PMC13499925; doi:10.1177/13872877261469556)
Supplement: Supplementary file 1 [file sj-docx-1-alz-10.1177_13872877261469556.docx]

**Supplemental Material**

**Early- and late-life brain reserve proxies and their interactions with education on cognitive outcomes in older adults: A population-based MRI study**

**Supplemental Table 1.** Characteristics of the study participants in the total sample and by late-life brain reserve levels

| **Characteristics** | **Total sample (n=1218)** | **Low late-life BR (n=609)** | | | **High late-life BR (n=609)** | | | ***p*** | | |
| --- | --- | --- | --- | --- | --- | --- | --- | --- | --- | --- |
| **Age (y), mean (SD)** | 69.36 (4.25) | 70.91 (4.09) | | | 67.82 (3.82) | | | <0.001 | | |
| **Female sex** | 710 (58.29) | 332 (54.52) | | | 378 (62.07) | | | 0.008 | | |
| ***APOE* ε4 allele^*^** | 184 (15.35) | 96 (16.05) | | | 88 (14.64) | | | 0.498 | | |
| **Education** |  |  | | |  | | | 0.039 | | |
| No formal schooling | 412 (33.83) | 189 (31.03) | | | 223 (36.62) | | |  | | |
| Any formal schooling | 806 (66.17) | 420 (68.97) | | | 386 (63.38) | | |  | | |
| **Occupation**^*^ |  |  | | |  | | | 0.611 | | |
| Farmers | 981 (80.67) | 487 (80.10) | | | 494 (81.25) | | |  | | |
| Non-framers | 235 (19.33) | 121 (19.90) | | | 114 (18.75) | | |  | | |
| **Cardiovascular factors** |  |  | | |  | | |  | | |
| Ever smoking | 385 (31.61) | 205 (33.66) | | | 180 (29.56) | | | 0.123 | | |
| Ever alcohol consumption^*^ | 471 (38.93) | 245 (40.56) | | | 226 (37.29) | | | 0.244 | | |
| BMI kg/m^2^, mean (SD) | 24.98 (3.51) | 24.98 (3.57) | | | 24.98 (3.46) | | | 0.981 | | |
| Hypertension^*^ | 816 (67.66) | 443 (73.59) | | | 373 (61.75) | | | <0.001 | | |
| Diabetes mellitus | 185 (15.19) | 105 (17.24) | | | 80 (13.14) | | | 0.046 | | |
| Dyslipidemia | 292 (23.97) | 147 (24.14) | | | 145 (23.81) | | | 0.893 | | |
| **Health conditions** |  |  | | |  | | |  | | |
| Stroke | 149 (12.23) | 93 (15.27) | | | 56 (9.20) | | | 0.001 | | |
| Coronary heart disease | 217 (17.82) | 116 (19.05) | | | 101 (16.58) | | | 0.261 | | |
| **Cognitive z-score, mean (SD)** | | | |  | | |  | | |  |
| Memory | 0.13 (0.85) | | -0.01 (0.85) | | | 0.27 (0.82) | | | <0.001 | |
| Verbal fluency | 0.12 (0.79) | | -0.02 (0.76) | | | 0.26 (0.79) | | | <0.001 | |
| Executive function | -0.01 (0.91) | | -0.07 (0.84) | | | 0.05 (0.98) | | | 0.020 | |
| Attention | 0.07 (0.86) | | -0.01 (0.81) | | | 0.16 (0.90) | | | 0.001 | |
| Global cognition | 0.08 (0.65) | | -0.03 (0.61) | | | 0.18 (0.68) | | | <0.001 | |
| **Brain MRI, mean (SD),** ml | | | |  | | |  | | |  |
| TIV | 1409.68 (127.29) | | 1413.34 (130.57) | | | 1406.02 (123.92) | | | 0.316 | |
| Brain parenchymal volume | 1061.33 (97.21) | | 1044.40 (96.13) | | | 1078.25 (95.40) | | | <0.001 | |
| WMH volume | 8.31 (10.93) | | 12.23 (13.27) | | | 4.40 (5.69) | | | <0.001 | |
| **MCI** | 274 (22.50) | | 161 (26.44) | | | 113 (18.56) | | | 0.001 | |
| **aMCI** | 232 (19.73) | | 141 (23.94) | | | 91 (15.50) | | | <0.001 | |
| **Non-aMCI** | 42 (4.26) | | 20 (4.12) | | | 22 (4.25) | | | 0.984 | |

Brain reserve proxies were derived from imaging data that were processed using the ComBat harmonization method to correct for batch effects across acquisition sites. Data were n (%), unless otherwise specified. BR: brain reserve; SD: standard deviation; *APOE*: apolipoprotein E; BMI: body mass index; TIV: total intracranial volume; WMH: white matter hyperintensities; MCI: mild cognitive impairment; aMCI: amnestic mild cognitive impairment; non-aMCI: non-amnestic mild cognitive impairment. ^*^Data were missing in 19 (1.56%) persons for *APOE* genotype, 2 (0.16%) for occupation, 8 (0.66%) for alcohol consumption, and 12 (0.99%) for hypertension. In subsequent analyses, the missing values were replaced with a dummy variable.

**Supplemental Table 2.** Association of early-life and late-life brain reserve proxies with cognitive performance (n=1218)

| **Brain reserve** | **β coefficient (95% confidence interval), cognitive z-score** | | | | |  |
| --- | --- | --- | --- | --- | --- | --- |
| **proxies** | **Memory** | **Verbal fluency** | **Executive function** | **Attention** | **Global cognition** | |
| **Model 1** |  |  |  |  |  | |
| **Early-life BR** |  |  |  |  |  | |
| Continuous | **-0.08 (-0.14, -0.02) ^†^** | 0.02 (-0.03, 0.07) | 0.05 (-0.01, 0.11) | **0.10 (0.04, 0.15)^†^** | 0.02 (-0.02, 0.06) | |
| Categorical |  |  |  |  |  | |
| Low | 0.00 (reference) | 0.00 (reference) | 0.00 (reference) | 0.00 (reference) | 0.00 (reference) | |
| High | -0.05 (-0.16, 0.06) | 0.01 (-0.09, 0.11) | 0.06 (-0.05, 0.16) | **0.14 (0.04, 0.24) ^†^** | 0.04 (-0.04, 0.12) | |
| **Late-life BR** |  |  |  |  |  | |
| Continuous | **0.14 (0.09, 0.19)^‡^** | **0.14 (0.10, 0.19)^‡^** | **0.08 (0.03, 0.12)^†^** | **0.10 (0.05, 0.14) ^‡^** | **0.12 (0.08, 0.15)^‡^** | |
| Categorical |  |  |  |  |  | |
| Low | 0.00 (reference) | 0.00 (reference) | 0.00 (reference) | 0.00 (reference) | 0.00 (reference) | |
| High | **0.21 (0.11, 0.31) ^‡^** | **0.24 (0.15, 0.33)****^‡^** | **0.10 (0.01, 0.20) ^∗^** | **0.16 (0.07, 0.25)^†^** | **0.18 (0.11, 0.24)^‡^** | |
| **Model 2** |  |  |  |  |  | |
| **Early-life BR** |  |  |  |  |  | |
| Continuous | **-0.08 (-0.14, -0.02)^†^** | 0.01 (-0.04, 0.07) | 0.04 (-0.02, 0.09) | **0.09 (0.03, 0.14) ^†^** | 0.01 (-0.03, 0.05) | |
| Categorical |  |  |  |  |  | |
| Low | 0.00 (reference) | 0.00 (reference) | 0.00 (reference) | 0.00 (reference) | 0.00 (reference) | |
| High | -0.04 (-0.15, 0.07) | 0.01 (-0.10, 0.11) | 0.05 (-0.06, 0.16) | **0.14 (0.03, 0.24) ^∗^** | 0.04 (-0.04, 0.11) | |
| **Late-life BR** |  |  |  |  |  | |
| Continuous | **0.14 (0.09, 0.19)^‡^** | **0.14 (0.09, 0.18)^‡^** | **0.07 (0.02, 0.12) ^†^** | **0.10 (0.05, 0.14) ^‡^** | **0.11 (0.07, 0.15)^‡^** | |
| Categorical |  |  |  |  |  | |
| Low | 0.00 (reference) | 0.00 (reference) | 0.00 (reference) | 0.00 (reference) | 0.00 (reference) | |
| High | **0.20 (0.10, 0.30) ^‡^** | **0.22 (0.13, 0.31)^‡^** | **0.09 (-0.01, 0.18)** | **0.15 (0.06, 0.25)^†^** | **0.16 (0.10, 0.23)^‡^** | |

Brain reserve proxies were derived from imaging data that were processed using the ComBat harmonization method to correct for batch effects across acquisition sites. Model 1 was controlled for age, sex, *APOE* genotype, and education. Model 2 was additionally adjusted for smoking, alcohol consumption, body mass index, hypertension, diabetes mellitus, hyperlipidemia, stroke, and coronary heart disease. BR: brain reserve. ^∗^*p* < 0.05, ^†^*p* < 0.01, ^‡^*p* < 0.001.

**Supplemental Table 3.** Association of early-life and late-life brain reserve proxies with mild cognitive impairment and subtypes (n=1218)

| **Brain reserve** | **Odd ratio (95% confidence interval)^a^** | | |
| --- | --- | --- | --- |
| **proxies** | **MCI (n=274)** | **aMCI (n=232)** | **non-aMCI (n=42)** |
| **Model 1** |  |  |  |
| **Early-life BR** |  |  |  |
| Continuous | 0.92 (0.77, 1.10) | 1.00 (0.83, 1.21) | **0.58 (0.38, 0.88)^∗^** |
| Categorical |  |  |  |
| Low | 1.00 (reference) | 1.00 (reference) | 1.00 (reference) |
| High | 0.91 (0.65, 1.27) | 1.00 (0.70, 1.41) | 0.55 (0.25, 1.21) |
| **Late-life BR** |  |  |  |
| Continuous | **0.74 (0.63, 0.86)^‡^** | **0.74 (0.63, 0.87)^‡^** | 0.74 (0.52, 1.04) |
| Categorical |  |  |  |
| Low | 1.00 (reference) | 1.00 (reference) | 1.00 (reference) |
| High | **0.63 (0.47, 0.85) ^†^** | **0.58 (0.42, 0.80) ^†^** | 1.05 (0.53, 2.08) |
| **Model 2** |  |  |  |
| **Early-life BR** |  |  |  |
| Continuous | 0.94 (0.79, 1.13) | 1.02 (0.84, 1.24) | **0.59 (0.38, 0.91)^∗^** |
| Categorical |  |  |  |
| Low | 1.00 (reference) | 1.00 (reference) | 1.00 (reference) |
| High | 0.90 (0.64, 1.26) | 0.99 (0.70, 1.42) | 0.51 (0.21, 1.22) |
| **Late-life BR** |  |  |  |
| Continuous | **0.73 (0.63, 0.86)^‡^** | **0.72 (0.61, 0.86)^‡^** | 0.80 (0.56, 1.16) |
| Categorical |  |  |  |
| Low | 1.00 (reference) | 1.00 (reference) | 1.00 (reference) |
| High | **0.64 (0.47, 0.87) ^†^** | **0.58 (0.42, 0.80) ^†^** | 1.23 (0.61, 2.48) |

Brain reserve proxies were derived from imaging data that were processed using the ComBat harmonization method to correct for batch effects across acquisition sites.

^a^The odds ratios (95% confidence intervals) of MCI were derived from binary logistic regression models and the odds ratios (95% confidence intervals) of aMCI and non-aMCI were derived from multinomial logistic regression models, in which individuals without MCI (n=944) were used as reference group in the modelling.

Model 1 was controlled for age, sex, *APOE* genotype, and education. Model 2 was additionally adjusted for smoking, alcohol consumption, body mass index, hypertension, diabetes mellitus, hyperlipidemia, stroke, and coronary heart disease. BR: brain reserve; MCI: mild cognitive impairment; aMCI: amnestic mild cognitive impairment; non-aMCI: non-amnestic mild cognitive impairment.

^∗^ *p* < 0.05, ^†^*p* < 0.01, ^‡^*p* < 0.001.

**Supplemental Figure 1.** Associations of early-life and late-life brain reserve proxies with memory (A & B), verbal fluency (C & D), executive function (E & F), and attention (G & H) z-scores from restricted cubic spline models (n=1218). The model was adjusted for age, sex, *APOE* genotype, education, smoking, alcohol consumption, body mass index, hypertension, diabetes, hyperlipidemia, stroke, and coronary heart disease. BR: brain reserve; *APOE*: apolipoprotein E gene.

**Supplemental Figure 2.** Associations of early-life and late-life brain reserve proxies which were processed using the ComBat harmonization method with memory (A & B), verbal fluency (C & D), executive function (E & F), and attention (G & H) z-scores from restricted cubic spline models (n=1218). The model was adjusted for age, sex, *APOE* genotype, education, smoking, alcohol consumption, body mass index, hypertension, diabetes, hyperlipidemia, stroke, and coronary heart disease. BR: brain reserve; *APOE*: apolipoprotein E gene.

**Supplemental Figure 3.** Association of joint brain reserve at early- and late-life and educational attainment with cognitive performance. Brain reserve proxies were derived from imaging data that were processed using the ComBat harmonization method to correct for batch effects across acquisition sites. Data were β coefficients (95% confidence intervals) of cognitive z-score associated with joint exposures to early- or late-life BR and educational attainment, derived from different models. Model 1 was controlled for age, sex, and *APOE* genotype. Model 2 was additionally adjusted for smoking, alcohol consumption, body mass index, hypertension, diabetes mellitus, hyperlipidemia, stroke, and coronary heart disease. CI: confidence interval; BR: brain reserve.

**Supplemental Figure 4.** Association of joint brain reserve at early- and late-life and educational attainment with MCI and its subtypes. Brain reserve proxies were derived from imaging data that were processed using the ComBat harmonization method to correct for batch effects across acquisition sites. Data were odds ratios (95% confidence intervals) of MCI or subtypes of MCI associated with joint exposures to early- or late-life BR and educational attainment, derived from different models. Model 1 was controlled for age, sex, and *APOE* genotype. Model 2 was additionally adjusted for smoking, alcohol consumption, body mass index, hypertension, diabetes mellitus, hyperlipidemia, stroke, and coronary heart disease. CI: confidence interval; BR: brain reserve; MCI: mild cognitive impairment; aMCI: amnestic mild cognitive impairment; non-aMCI: non-amnestic mild cognitive impairment.

**Supplemental Figure 5.** Interaction of educational attainment and late-life brain reserve proxy on cognitive performance. Brain reserve proxies were derived from imaging data that were processed using the ComBat harmonization method to correct for batch effects across acquisition sites. Plots illustrate the significant moderating role of educational attainment in the associations between late-life BR and (A) executive function, (B) attention, and (C) global cognition. Educational attainment was dichotomized as high (any formal school education; green dotted line) or low (no formal school education; red dotted line). Shaded areas denote 95% confidence intervals. Analyses were adjusted for age, sex, *APOE* genotype, smoking, alcohol consumption, body mass index, hypertension, diabetes mellitus, hyperlipidemia, stroke, and coronary heart disease. BR: brain reserve; *APOE*: apolipoprotein E gene.
